# Next generation viscoelasticity assays in cardiothoracic surgery: Feasibility of the TEG6s system

**DOI:** 10.1371/journal.pone.0209360

**Published:** 2018-12-20

**Authors:** Gabor Erdoes, Hannes Schloer, Balthasar Eberle, Michael Nagler

**Affiliations:** 1 Department of Anesthesiology and Pain Medicine, Inselspital, Bern University Hospital, University of Bern, Bern, Switzerland; 2 Department of Hematology and Central Hematology Laboratory, Inselspital, Bern University Hospital, University of Bern, Bern, Switzerland; 3 Department of Clinical Research, University of Bern, Bern, Switzerland; IRCCS Policlinico S.Donato, ITALY

## Abstract

**Background:**

Viscoelastic near-patient assays of global hemostasis have been found useful and cost-effective in perioperative settings. Shortcomings of current systems include substantial laboratory intensity, user-dependent reproducibility, relatively large sample volumes, sensitivity to ambient vibration and limited comparability between techniques and devices. The aim of this study was to assess feasibility of a new, resonance-based viscoelastic whole blood methodology (TEG6s) in cardiac surgery with cardiopulmonary bypass (CPB) and to compare the parameters this system produces with the ROTEM delta system and standard coagulation tests.

**Methods:**

In a prospective evaluation study, twenty-three consecutive cardiac surgery patients underwent hemostasis management according to current guidelines, using the ROTEM delta system and standard coagulation tests. Blood samples were collected prior to CPB before anesthetic induction (pre-CPB), during CPB on rewarming (CPB), and 10 minutes after heparin reversal with protamine (post-CPB). ROTEM and standard coagulation test results were compared with TEG6s parameters, which were concurrently determined using its multi-channel microfluidic cartridge system.

**Results:**

TEG6s provided quantifiable results pre-CPB and post-CPB, but only R (clotting time) of CKH (kaolin with heparinase) was measurable during CPB (full heparinization). Spearman’s correlation coefficient (r_s_) was 0.78 for fibrinogen levels and MA CFF (functional fibrinogen). Correlation of several TEG6s parameters was good (0.77 to 0.91) with MCF FIBTEM, and poor (<0.56) with prothrombin time and activated partial thromboplastin time (<0.44). R_s_ with platelet count was moderate (0.70, MA CK; 0.73, MA CRT). Accuracy of MA CFF for detection of fibrinogen deficiency < 1.5 g/L was high (ROC-AUC 0.93).

**Conclusions:**

The TEG6s system, which is based on resonance viscoelastic methodology, appears to be feasible for POC hemostasis assessment in cardiac surgery. Its correlations with standard coagulation parameters are quite similar to those of ROTEM and there is good diagnostic accuracy for fibrinogen levels lower than 1.5 g/L. During full heparinization, TEG6s testing is limited to R measurement. Larger studies are needed to assess superiority over other POC systems.

## Introduction

Cardiac surgery with cardiopulmonary bypass (CPB) is frequently complicated by coagulopathic bleeding, which may require transfusion of allogenic blood products and may thus worsen outcome. [1] Current guidelines recommend monitoring of hemostasis using viscoelastic hemostatic assays (VHA) in conjunction with coagulation management algorithms, with the aim to reduce patients`transfusion exposure. [2–4] Point-of-care (POC) coagulation monitoring with VHA is increasingly used to guide administration of factor concentrates, especially in response to severe perioperative bleeding in cardiac surgery. [5–7]

Compared to standard laboratory coagulation testing, VHA provides comprehensive in vitro evaluation of patients’ functional hemostasis rapidly enough to guide emergency treatment. [8,9] During and after CPB, graphically displayed VHA results allow intuitive interpretation and proposal of specific interventions (fibrinogen supplementation, additional protamine administration) to improve coagulation rapidly. [10–13]

Established VHA instruments such as thromboelastography (TEG, Haemonetics Inc., Braintree, MA, USA) or thromboelastometry (ROTEM TEM International GmbH, Munich, Germany) assess hemostasis properties by measuring shear forces between a pin and a blood-filled cup. Oscillation is either generated by the pin or by the cup. [14] However, current VHA are associated with important limitations—in particular, limited consistency. [10,15,16] These systems are also bulky and sensitive to vibration. In addition, due to manual sampling and mixing, skilled technicians are needed to perform frequent calibration and cleaning procedures. [17]

To overcome these limitations, next-generation viscoelastic devices have been developed. Haemonetics recently introduced a fully automated thromboelastograph, the TEG6s (Haemonetics Inc., Braintree, MA, USA).[18] The instrument assesses whole blood coagulation properties using the resonance method, which means that a blood sample within a four-channel self-contained microfluidics cartridge is exposed to a sinusoidal motion range (20–500 Hz). As clotting proceeds, clot-strength-specific resonance frequencies are detected by a photodetector and converted into TEG-equivalent units. The latter are used to generate TEG tracings, which are illustrating the viscoelastic change of the blood sample in real time. [18]

A faster test setup, which is portable, easy to maintain and does not require manual pipetting and mixing would seem to be a promising innovation, allowing efficient POC coagulation monitoring in patients undergoing cardiac surgery. [19] Considering these advantages, TEG6s is aiming to replace established VHA systems. However clinical experience with TEG6s is limited and randomized, controlled evaluations are rare.

The aim of this study was to assess the feasibility of the TEG6s system in cardiothoracic surgery. We studied the distribution of TEG6s results in the course of surgery, calculated reference ranges, determined the correlation between TEG6s results and established laboratory parameters including thromboelastometry (ROTEM delta), and calculated the accuracy with regard to fibrinogen deficiency.

## Methods

### Study design, setting and patients

This prospective observational study was conducted between July 11 and August 12, 2016, at a tertiary care hospital. We included twenty-three consecutive adult patients scheduled for elective cardiovascular surgery with CPB. We observed the TEG6s measurements in the course of cardiac surgery and explored associations between TEG6s measurements and established laboratory parameters. Exclusion criteria were emergency surgery, age less than 18 years or absence of informed consent. The study was approved by the Cantonal Ethics Committee (#18–00415) and all patients gave their written informed consent. The manuscript adheres to reporting guidelines on diagnostic accuracy (STARD).

### Collection of data

Baseline characteristics of the patients (age, sex, body mass index, presence of concomitant disorders, and treatment with anticoagulants or antiaggregants) were collected using the electronic patient record and transferred to a pseudonymized, encrypted database. Details of the perioperative management were transferred from the anesthesia protocol, which included type and details of surgery, and treatment with anticoagulants, coagulation factor concentrates and allogeneic blood products. Laboratory tests were performed at three specified time points—prior to CPB before anesthetic induction (pre-CPB), during CPB on rewarming (CPB) and 10 minutes after heparin reversal with protamine (post-CPB)—and transferred to the database mentioned above.

### Perioperative management of anesthesia, CPB technique, and hemostasis

All patients received general anesthesia. CPB was performed with two different systems, both without heparin-coated components. For coronary artery bypass grafting (CABG) the minimized extracorporeal circulation (MiECC, Maquet, Rastatt, Germany) was used. A conventional extracorporeal circulation (CECC, Maquet) was used for all non-CABG cardiothoracic surgeries. Prior to aortic cannulation an initial bolus of heparin was administered (CECC: 500 IU/kg ideal body weight (IBW); MiECC: 400 IU/kg IBW). Additional boluses of heparin were administered to maintain a target kaolin-activated clotting time (ACT, Activated Coagulation Timer ACT II, Medtronic Minneapolis, USA) of > 480 seconds with MiECC and > 600 seconds with CECC. Following CPB weaning, anticoagulation was antagonized with protamine chloride in a 1:1 ratio to the initial heparin bolus, targeting a normal post-interventional ACT value. Moderate hypothermia was applied in all cases except for patients with hypothermic cardiocirculatory arrest (HCA, 28 °C). Routinely all patients received a single bolus of tranexamic acid (dose: 10 mg/kg IBW), followed by a continuous infusion (5 mg/kg IBW/h) until sternal closure. In patients requiring CECC, shed blood was either filtered and directly conducted to the cardiotomy reservoir during CPB or underwent filtration and cell salvage (Autolog, Medtronic Inc., Minneapolis, MN, USA) after CPB. Remaining blood in the CPB circuit was either cell salvaged and retransfused (in CECC) or directly reinfused before heparin neutralization (in MiECC).

Administration of blood products and hemostatic agents was followed by the in-house patient blood management (PBM) protocol which is a transfusion algorithm based on existing literature incorporating tests results obtained from point-of-care devices (e.g. ROTEM).

Fibrinogen concentrate has been administered in case of persistent non-surgical bleeding (> 150–200 ml/hr) after weaning from CPB and heparin neutralization if at the same time a hypofibrinogenemia was present in ROTEM (FIBTEM A10 < 10 mm) or in the conventional test (Clauss fibrinogen < 1.5 g/l). The target range of plasma fibrinogen was FIBTEM A10 > 12 mm or Clauss fibrinogen 1.5–2.0 g/l according to the recent guidelines set by the European Society of Anesthesiology. [2]

### Collection and handling of samples

Citrated blood samples were drawn under standardized conditions using citrated plastic syringes (0.106 mol/L; Monovette, Sarstedt, Nuembrecht, Germany) at the above mentioned three specified time points. An established and implemented protocol was followed ensuring adequate preanalytic conditions; samples for coagulometric tests were centrifuged for 15 minutes at 1500 g. The following tests were performed simultaneously within 30 minutes: TEG6s, ROTEM delta, fibrinogen (Clauss method), prothrombin time (PT; Quick percent), activated partial thromboplastin time (aPTT), as well as platelet count.

### Determination of TEG6s results

A TEG6s was implemented (Haemonetics Inc., Braintree, MA, USA) and analyses were conducted within 30 minutes according to the manufacturer’s instructions. [17] In contrast to previous devices, no handling of reagents was necessary. The cartridges contained all required reagents in four microfluidic channels: kaolin in the CK-channel, kaolin and tissue factor in the CRT-channel, kaolin and heparinase in the CKH-channel, as well as kaolin and abciximab in the CFF-channel. A small amount of citrated whole blood (0.3 ml) was pipetted into the cartridge, the carriage was placed in the device, and determination of the assays was initiated automatically. An optical detector measured the movements of a blood meniscus exposed to a fixed vibration frequency range and results were transformed to the TEG readout. More details are described elsewhere. [17] For the purpose of our study, we recorded the clotting time (R) and maximum clot strength (MA).

### Determination of ROTEM measurements and other laboratory tests

Platelet counts were determined using a Coulter Counter LH750 (Beckman-Coulter Inc., Nyon, Switzerland).

Plasma levels of fibrinogen were measured according to the Clauss method using Multifibren U reagent (Siemens Healthcare Diagnostics, Marburg, Germany). [20] Prothrombin time was determined using Dade Innovin (Siemens Healthcare Diagnostics, Marburg, Germany) and aPTT was measured using Pathrombin SL (Siemens Healthcare Diagnostics, Marburg, Germany). All analyses were run on a CS5100 coagulometer (Siemens Healthcare Diagnostics, Marburg, Germany). Thromboelastometry analysis was performed on a ROTEM delta analyzer according to the manufacturer’s instructions (ROTEM delta; Tem International GmbH, Munich, Germany) with multiple-test reagents. INTEM, EXTEM, FIBTEM, and HEPTEM test were conducted in citrated whole blood following a including the 5-minute pre-heating procedure.

### Statistical analysis

The correlation of TEG6s parameters with established laboratory parameters was considered as main analysis. We were aiming for a minimum correlation of 0.55, a power of 0.8, and an α of 0.05 (two-tailed). Following an established formula, [21], 22 patients were estimated.

Median and 5^th^ to 95^th^ percentiles or frequency and percentages were reported as appropriate. Spearman’s rank correlation coefficient (r_s_) was used to describe associations between TEG6s results and established laboratory parameters. Wilcoxon matched-pairs signed rank test was used to test differences of TEG6s results between pre-CPB and post-CPB measurements. A receiver-operating characteristics curve (ROC) was constructed and area under the curve (AUC) was calculated to explore the accuracy of MA CFF and MA CKH for fibrinogen deficiency. All analyses were performed using the Stata 13.1 statistics software package (StataCorp. 2013. Stata Statistical Software: Release 13. College Station, TX, USA); Figures were created using GraphPad Prism version 6.00 for Mac OS X (GraphPad Software, La Jolla, CA, USA).

## Results

### Baseline characteristics

Among the 23 patients included, seven (30%) underwent composite graft implantation, five (22%) received coronary artery bypass surgery, two (9%) valve surgery, four (17%) combined surgery and five (22%) other procedures. Median age was 67 years and 5 patients were female (22%). Eleven patients (48%) received neither autologous blood products (e.g., packed red blood cells [PRBC], fresh frozen plasma [FFP] or platelet concentrate [PLT]) nor fibrinogen or other factor concentrates during and after surgery.

Detailed baseline characteristics as well as surgical details are presented in Table 1.

**Table 1:** Baseline characteristics.

Age, years	67 (26/78)
Female sex	5 (22)
BMI, kg/m^2^	25 (15/38)
Concomitant disorders:	
Hypertension	15 (65)
COPD	4 (17)
Diabetes mellitus	1 (4)
CKD	10 (43)
Pre-operative treatment with antithrombotic drugs:	
Antiaggregant drugs	15 (65)
Anticoagulants	5 (22)
Surgical procedure:	
CABG	5 (22)
Valve surgery	2 (9)
Valve surgery and CABG	4 (17)
Thoracic aorta repair	2 (9)
Composite Graft	7 (30)
LVAD	1 (4)
Conduit	1 (4)
David procedure	1 (4)
Details of procedure	
Procedural time, min	286 (204/508)
CPB, min	135 (52/214)
HCA	8 (35)
Anticoagulants, coagulation factors, and blood products:	
Heparin, kU	40 (22/80)
Protamine, kU	40 (25/120)
PRBC, U	0 (0/8)
FFP, U	0 (0/5)
PLT, U	0 (0/2)
Autologous blood, ml	480 (0/1340)
Application of hemostatic agents:	
Fibrinogen concentrate	9 (39)

Data correspond to median (minimum/ maximum) or number (percent), where appropriate. Pre-operative anticoagulants were given less than five days prior to intervention. Abbreviations: BMI, body mass index; COPD, chronic obstructive pulmonary disease; CKD, chronic kidney disease, def. as serum creatinine > 150 mmol/l; CABG, coronary arterial bypass grafting; LVAD, left ventricular assist device; CPB, cardiopulmonary bypass; HCA, hypothermic cardiocirculatory arrest; PRBC, packed red blood cell concentrates; FFP, fresh frozen plasma; PLT, platelet concentrate; U, units; kU, kilo units

### Distribution of TEG6 parameters in the course of surgery

**Fig 1:**
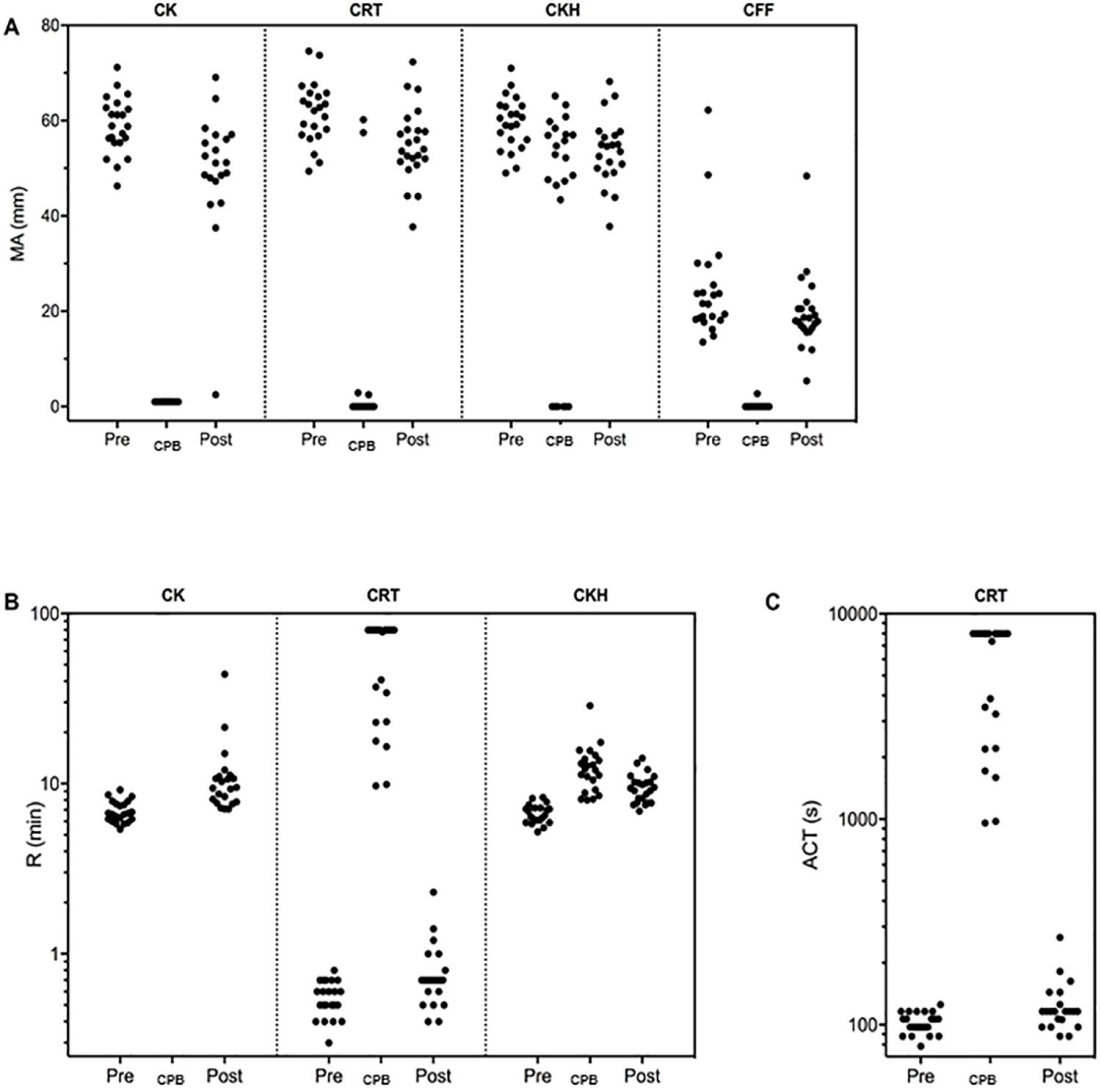
Pattern of TEG6s parameters in the course of cardiothoracic surgery. (A) reaction time (R), (B) maximum amplitude (MA), and (C) activated clotting time equivalent (ACT) values are shown before cardiopulmonary bypass (CPB; Pre), during (CPB) and after (Post). Abbreviations: CK, Kaolin TEG; CRT, rapid TEG; CKH, TEG with heparinase; CFF, TEG functional fibrinogen.

Median and 5^th^ to 95^th^ percentiles (corresponding to reference ranges) of TEG6s parameters in the course of surgery are reported in Table 2; their distribution is shown in Fig 1. Results in the measurable range were observed pre-CPB and post-CPB for most patients and parameters and for CPB in the case of R CKH. However, no results were determinable during CPB (full heparinization) for MA CK, MA CRT, MA CFF, or R CK (partly determinable for MA CKH and R CRT). Consequently, determination of functional fibrinogen during CPB using the TEG6s’s CFF assay was not possible in our population. In addition, TEG6s on-pump ACT measurements (CRT Channel, R time) showed a wide distribution, with 5^th^ and 95^th^ percentile values of 958 seconds and 7347 seconds, respectively (Table 2), making reliable heparin monitoring using the TEG6s device during CPB unlikely.

**Table 2:** Distribution of TEG6s parameters in the course of cardiothoracic surgery (reference ranges).

Parameter	Pre-CPB		CPB		Post-CPB		Probability^+^
	Median (5^th^, 95^th^ percentile)*	Not clottable (number, percent)	Median (5^th^, 95^th^ percentile)*	Not clottable (number, percent)	Median (5^th^, 95^th^ percentile)*	Not clottable (number, percent)	
MA CK (mm)	58.9 (50.2, 67.4)	1 (4.3%)	N/A	23 (100%)	51.2 (20.0, 66.9)	3 (13.0%)	p = 0.003
R CK (min)	6.7 (5.8, 8.6)	1 (4.3%)	N/A	23 (100%)	9.5 (7.1, 21.4)	1 (4.4%)	p<0.0001
MA CRT (mm)	62.5 (51.2, 73.7)	1 (4.3%)	30.2 (2.5, 60.2)	19 (82.6%)	54.0 (44.1, 67.2)	0	p = 0.01
R CRT (min)	0.6 (0.4, 0.7)	1 (4.3%)	23 (9.7, 78.0)	13 (56.5%)	0.7 (0.4, 1.4)	0	p = 0.005
ACT CRT (sec)	102.0 (87.9, 116.0)	1 (4.3%)	2202.2 (957.9, 7347.0)	13 (56.5%)	116.0 (87.9, 181.5)	0	p = 0.006
MA CKH (mm)	59.9 (50.0, 67.4)	1 (4.3%)	55.8 (43.4, 65.2)	6 (26.1%)	54.6 (43.9, 65.2)	2 (8.7%)	p = 0.01
R CKH (min)	6.5 (5.5, 8.2)	2 (8.7%)	12.1 (8.1, 17.5)	1 (4.3%)	9.4 (7.5, 13.2)	0	p<0.0001
MA CFF (mm)	21.6 (14.8, 48.6)	1 (4.3%)	2.6 (2.5, 2.7)	21 (91.3%)	18.0 (11.9, 28.3)	0	p = 0.09

Abbreviations: R = reaction time, MA = maximum amplitude, ACT = activated clotting time equivalent, CK = Kaolin TEG, CRT = rapid TEG, CKH = TEG with heparinase, CFF = TEG functional fibrinogen, pre-CPB = values before cardiopulmonary bypass, CPB = during cardiopulmonary bypass, post-CPB = after cardiopulmonary bypass;

* corresponding to reference ranges;

^+^ pre-CPB vs. post-CPB (Wilcoxon matched-pairs signed rank test)

### Correlation of TEG6 results with established laboratory parameters

Correlation of TEG6s results with fibrinogen levels varied between 0.68 (MA CKH) and 0.80 (MA CK; MA CRT). Correlation was higher with regard to MCF FIBTEM (ROTEM; functional fibrinogen), 0.77 in the case of MA CK, and 0.91 in the case of MA CFF. Lower correlation coefficient were observed with regard to PT, aPTT and platelet count. Details are given in Table 3, and scatter plots are illustrated in S1, S2 and S3 Figs.

**Table 3:** Correlation of TEG6s results with established coagulation parameters, platelet count, and thromboelastometry results.

TEG6s parameters	Fibrinogen	PT (Quick %)	aPTT	Platelet count	MCF FIBTEM	CT INTEM	CT EXTEM	MCF HEPTEM
	Spearman’s rank correlation coefficient r_s_ (95% CI)							
R CK*			0.41 (0.10, 0.64)			0.39 (0.10, 0.62)		
MA CK*	0.80 (0.65, 0.89)		-0.44 (-0.67, -0.14)	0.70 (0.49, 0.84)	0.77 (0.60, 0.87)			
R CRT*		-0.56 (-0.74, -0.31)					0.51 (0.25, 0.70)	
MA CRT*	0.80 (0.65, 0.89)	0.48 (0.20, 0.68)		0.73 (0.55, 0.85)	0.85 (0.74, 0.92)			
ACT CRT*			0.35 (0.04, 0.60)					
MA CFF*	0.78 (0.63, 0.88)				0.91 (0.84, 0.95)			
MA CKH^+^	0.68 (0.30, 0.87)				0.83 (0.55, 0.94)			0.85 (0.58, 0.95)

Abbreviations: R = reaction time, MA = maximum amplitude, ACT = activated clotting time equivalent, CK = Kaolin TEG, CRT = rapid TEG, CKH = TEG with heparinase, CFF = TEG functional fibrinogen,

* values before and after cardiopulmonary bypass were considered,

^+^ values during cardiopulmonary bypass were considered

### Accuracy of TEG6 results with regard to hypofibrinogenemia

**Fig 2:**
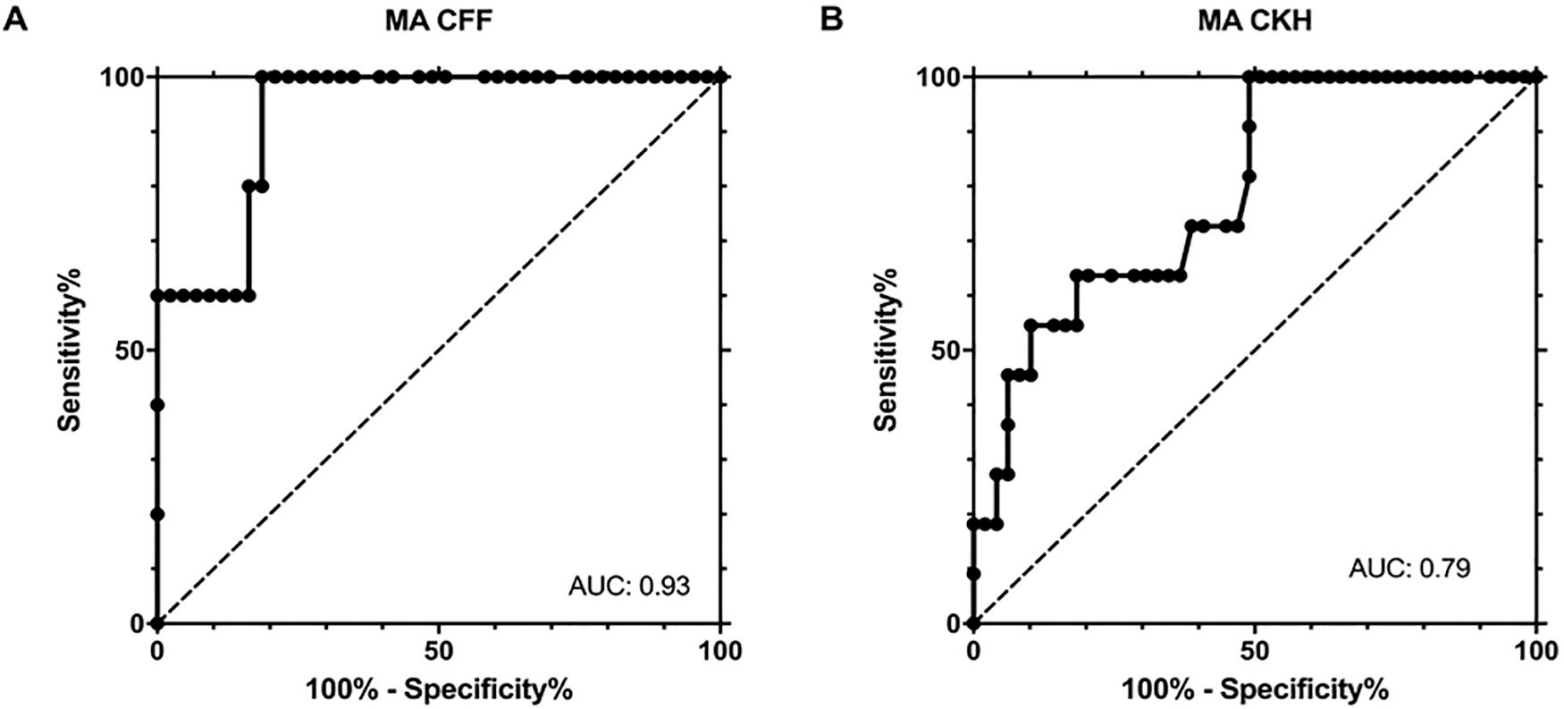
Accuracy of TEG6s results for hypofibrinogenemia (<1.5 g/L). ROC curves of (A) MA CFF and (B) MA CKH are shown. (A) Area under the ROC curve (AUC) of MA CFF was 0.93 (95% CI 0.84, 1.00). At a threshold of 17.2 mm sensitivity was 100% (95% CI 47.8, 100.0) and specificity 81.4% (95% CI 66.6, 91.7); (B) AUC of MA CKH 0.79 (95% CI 0.66, 0.93). At a threshold of 57.3 mm sensitivity was 100% (95% CI 71.5, 100.0) and specificity was 51% (95% CI 36.3, 65.6).

The area under the receiver-operating characteristics curve for the detection of fibrinogen levels below 1.5 g/L was 0.93 in the case of MA CFF and 0.79 in the case of MA CKH. Thresholds of 17.2 mm for MA CFF and of 57.3 mm for MA CKH yielded the best sensitivity and specificity (Fig 2).

## Discussion

The TEG6s was rapidly implemented for perioperative management in cardiothoracic surgery and could be handled reliably without extensive training measures. Whereas interpretable results were obtained in most patients in the pre-CPB and post-CPB period, few parameters could be measured during full heparinization (R CKH). TEG6s parameters correlated adequately with fibrinogen and functional fibrinogen (MCF FIBTEM) and the accuracy of MA CFF for detecting fibrinogen levels below 1.5 g/L was high.

Few previous studies have investigated the TEG6s device. Gurbel studied 300 patients undergoing coronary revascularization in a first validation study in 2015. TEG6s measurements demonstrated high precision as well as a strong correlation with the results of the established TEG5000. In contrast to our study, those authors did not report any influence of heparin administration on TEG6s parameters. [17] Moynihan investigated correlations between heparin dose and alterations of TEG6s parameters (R times for CK and CKH) during pediatric extracorporeal life support. Administering a smaller heparin dose (50 U/kg bolus, 20 IU/kg/h maintenance targeting ACT of 300 sec) as in our study, Moynihan could measure feasible values for TEG6s R time in the CK channel during CPB. [22] Furthermore, there has been research into the influence of non-vitamin K oral anticoagulants (NOACs) or the effect of sample storage duration on TEG6s parameters.

Comparability of these study results to our findings on CPB is limited, because blood samples in both studies were drawn from healthy blood donors without high dose heparinization. [18,19] Finally, a recent study investigated TEG6s vibration resistance by placing it on a flatbed platelet agitator. In contrast to their examination, we neither exposed our TEG6s device to such vibration nor used blood samples from healthy donors. [23]

A strength of our study is that we looked at several important feasibility aspects of TEG6s in the context of cardiothoracic surgery in clinical practice. We also had important limitations, however. As a feasibility study, we had included a limited number of patients. Thus, the confidence intervals are large and the estimations not very precise. We were also not able to associate the TEG6s measurements with clinical outcomes. Moreover, our results should be confirmed in other settings as well. While TEG6s appears to be of limited value during CPB, the high operator convenience and adequate correlation with fibrinogen levels (as well as functional fibrinogen) suggests a potential improvement of perioperative hemostasis management. Future studies including more patients must establish the benefit in different settings before widespread implementation.

In conclusion, the new TEG6s device utilizing the resonance viscoelastic test system appears to be an easy-to-use point-of-care device which may improve procedures in the perioperative setting. Although correlation with established laboratory parameters is adequate, only a few measurements could be performed during full heparinization. Larger studies would determine the diagnostic accuracy in detail.

## Supporting information

S1 Fig Correlation of CFF and CKH parameters with conventional coagulation parameters, platelet count as well as ROTEM results: Maximum amplitude (MA), maximum clot firmness (MCF), CK, Kaolin TEG; CRT, rapid TEG; CKH, TEG with heparinase; CFF, TEG functional fibrinogen. FIBTEM, Rotem with tissue factor and cytochalasin A; HEPTEM, Rotem with partial thromboplastin phospholipid and heparinase.
(TIF)

S2 Fig Correlation of CK, CKH, CRT, CFF parameters with conventional coagulation parameters, as well as ROTEM results following cardiopulmonary bypass surgery: Maximum amplitude (MA), maximum clot firmness (MCF), CK, Kaolin TEG; CRT, rapid TEG; CKH, TEG with heparinase; CFF, TEG functional fibrinogen. FIBTEM, Rotem with tissue factor and cytochalasin A; HEPTEM, Rotem with partial thromboplastin phospholipid and heparinase; INTEM, Rotem with partial thromboplastin phospholipid, EXTEM, Rotem with tissue factor.
(TIF)

S3 Fig Correlation of CK and CRT parameters with conventional coagulation parameters, platelet count as well as ROTEM results following cardiopulmonary bypass surgery: Maximum amplitude (MA), maximum clot firmness (MCF), CK, Kaolin TEG; CRT, rapid TEG, FIBTEM, Rotem with tissue factor and cytochalasin A. (TIF)
