# Supplementary figures and images for: Next generation viscoelasticity assays in cardiothoracic surgery: Feasibility of the TEG6s system
Source: PLoS One. 2018 Dec 20;13(12):e0209360. doi: 10.1371/journal.pone.0209360 (PMC6301661; doi:10.1371/journal.pone.0209360)

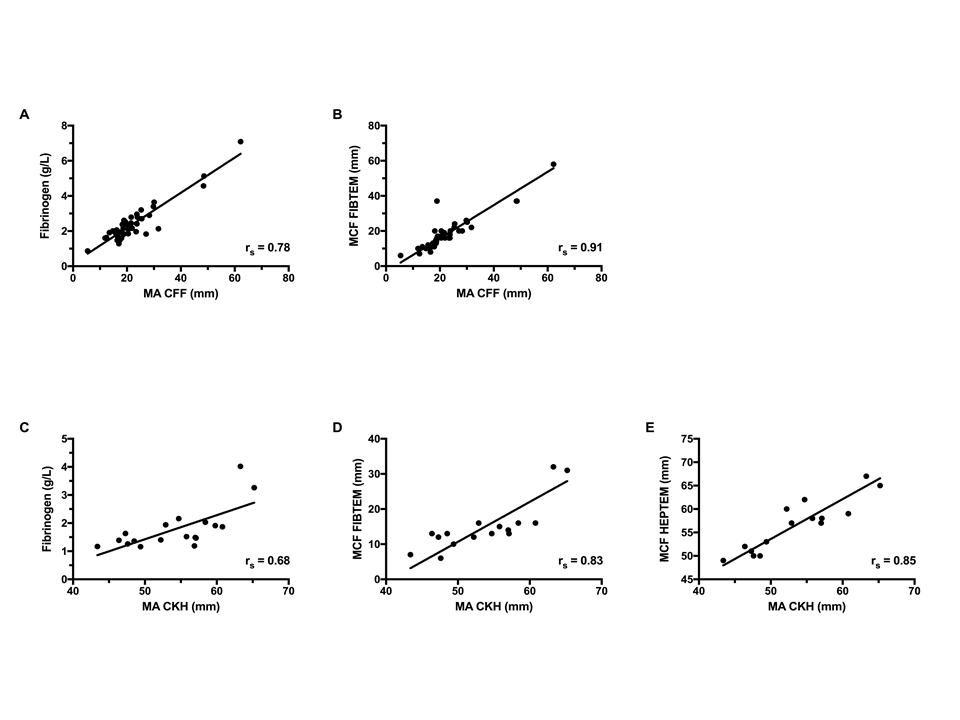

Supplement: S1 Fig — FIBTEM, Rotem with tissue factor and cytochalasin A; HEPTEM, Rotem with partial thromboplastin phospholipid and heparinase. (TIF) [file pone.0209360.s001.TIF]

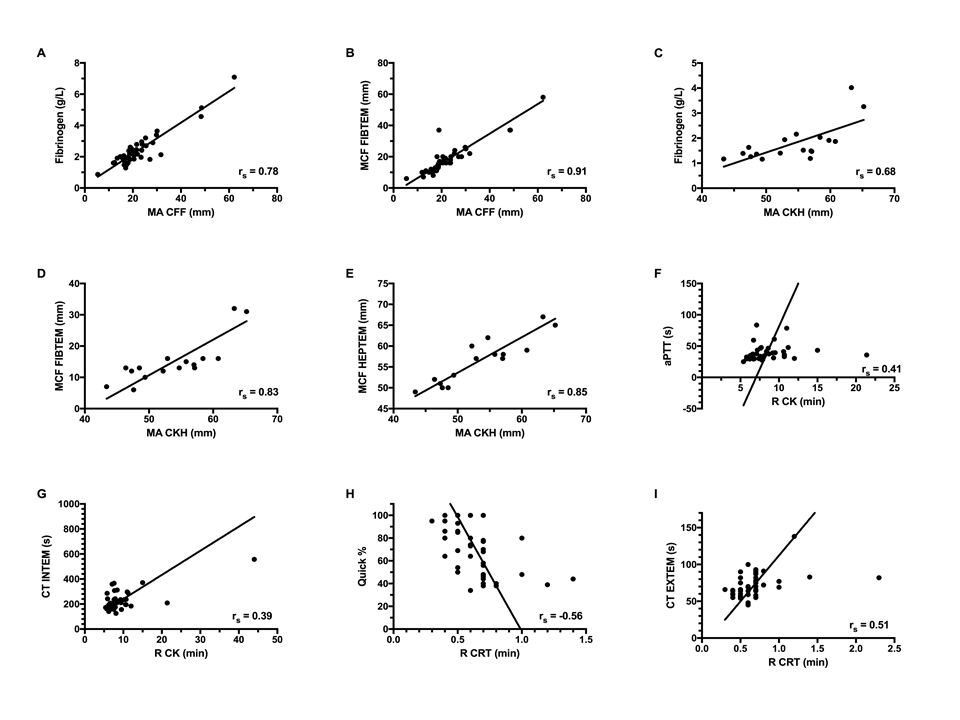

Supplement: S2 Fig — FIBTEM, Rotem with tissue factor and cytochalasin A; HEPTEM, Rotem with partial thromboplastin phospholipid and heparinase; INTEM, Rotem with partial thromboplastin phospholipid, EXTEM, Rotem with tissue factor. (TIF) [file pone.0209360.s002.TIF]

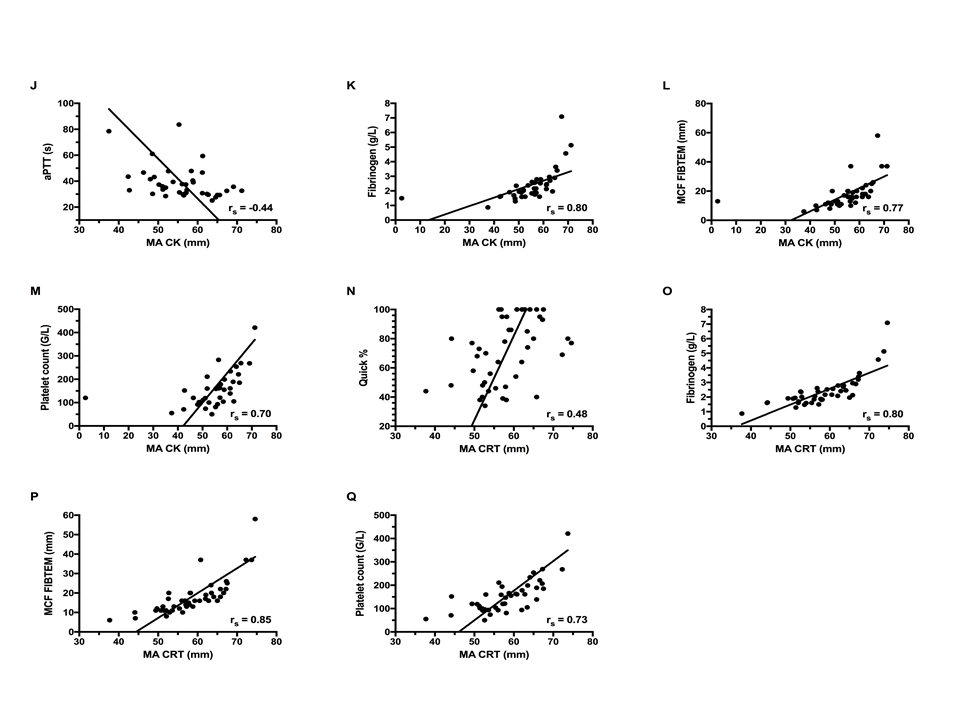

Supplement: S3 Fig — (TIF) [file pone.0209360.s003.TIF]
